# Functional Characterization of Porcine NK-Lysin: A Novel Immunomodulator That Regulates Intestinal Inflammatory Response

**DOI:** 10.3390/molecules26144242

**Published:** 2021-07-13

**Authors:** Qian Lin, Qingqing Fu, Daiwen Chen, Bing Yu, Yuheng Luo, Zhiqing Huang, Ping Zheng, Xiangbing Mao, Jie Yu, Junqiu Luo, Hui Yan, Jun He

**Affiliations:** 1 Institute of Animal Nutrition, Sichuan Agricultural University, Chengdu 611130, China; linqian@stu.sicau.edu.cn (Q.L.); fqq0679@163.com (Q.F.); chendwz@sicau.edu.cn (D.C.); ybingtian@163.com (B.Y.); luoluo212@126.com (Y.L.); z.q.huang@163.com (Z.H.); zpind05@163.com (P.Z.); acatmxb2003@163.com (X.M.); jerryyujie@163.com (J.Y.); luojunqiu@163.com (J.L.); yan.hui@sicau.edu.cn (H.Y.); 2 Key Laboratory of Animal Disease-Resistant Nutrition, Chengdu 611130, China

**Keywords:** antimicrobial peptide, hemolytic activity, MIC, Porcine NK-Lysin

## Abstract

Porcine NK-Lysine (PNKL) is a new antimicrobial peptide (AMP) identified in the small intestine. In this study, PNKL protein was obtained through heterologous expression in *Escherichia coli* and was estimated by SDS-PAGE at 33 kDa. The antibacterial activities of PNKL were determined using various bacterial strains and showed broad-spectrum antimicrobial activity against Gram-negative and Gram-positive bacteria. Furthermore, *E. coli* K88-challenged IPEC-J2 cells were used to determine PNKL influences on inflammatory responses. Hemolytic assays showed that PNKL had no detrimental impact on cell viability. Interestingly, PNKL elevated the viability of IPEC-J2 cells exposure to *E. coli* K88. PNKL significantly decreased the cell apoptosis rate, and improved the distribution and abundance of tight junction protein ZO-1 in IPEC-J2 cells upon *E. coli* K88-challenge. Importantly, PNKL not only down regulated the expressions of inflammatory cytokines such as the IL-6 and TNF-α, but also down regulated the expressions of NF-κB, Caspase3, and Caspase9 in the *E. coli* K88-challenged cells. These results suggest a novel function of natural killer (NK)-lysin, and the anti-bacterial and anti-inflammatory properties of PNKL may allow it a potential substitute for conventionally used antibiotics or drugs.

## 1. Introduction

Enterotoxigenic *Escherichia coli* (ETEC) is one of the main pathogens causing diarrhea in newborn neonatal animals, which causes huge economic losses in the livestock industry [1]. Infection of a newborn with ETEC frequently associate with severe watery diarrhea, rapid dehydration, and even death [2]. ETEC binds to interaction with the small intestinal mucosa by adhering to epithelial cells and then multiplies in the intestine, which subsequently produces enterotoxins that act on the intestine and lead to the secretion of large amounts of fluid in the small intestinal liquid, electrolytes, and causing diarrhea [3]. Currently, antibiotics and other chemical drugs are widespread therapeutic regimen to treat the ETEC infection. However, the long-term or over-dose utilization of complex antibiotics not only causes pathogen resistance and mutation, but also weakens the role of antibiotics or intensely accumulates in the body, thereby inducing gastrointestinal microbiota disorder, and causing a variety of diseases [4,5,6]. Therefore, substitutes for conventionally used antibiotics have attracted extensive research attention worldwide.

The most widely studied alternatives include probiotics, prebiotics, enzymes, acidifiers, plant extracts, and nutraceuticals such as copper and zinc. Antimicrobial peptides (AMPs) are clusters of immune-related peptides/proteins that can protect the host from pathogen infections [7,8]. It is an essential part of the internal defense of innate immunity of animal and plant kingdom and widespread in natures [9]. Antimicrobial peptides have a broad spectrum of antibacterial activities, as they can kill bacteria by permeating their membranes [10,11]. Although the antibacterial activity of AMPs against a specific pathogen is normally weaker than several conventionally used antibiotics, one of their major advantages is the ability to kill multidrug-resistant bacteria at relatively low concentrations [12]. Moreover, AMPs may involve multiple mechanism of bacteriostasis [13]. In addition to their antibacterial activity, AMPs have also been found to act as novel agents against bacterial biofilms and immunomodulators of the host immune system. For instance, catestatin has a close interaction with the neuroendocrine system and the immune system, which can activate human mast cells [14]. Ocellatin peptides is believed to inhibit the biofilm formation or to eradicate established ones [15]. Moreover, AMPs can regulate the secretion of inflammatory factors. For instance, the alpha-melanocyte stimulating hormone (α-MSH) reduces the concentration of proinflammatory mediators through the induction of cyclic adenosine monophosphate (cAMP) and inhibition of the nuclear factor κβ (NF-κβ), thus protecting the brain and peripheral organs from inflammatory disorders [16].

Natural killer (NK)-lysin, a member of saponin-like protein family [17], is a 9-kDa cationic protein which originally isolated from pig intestinal tissue and used as an effector peptide of cytotoxic T lymphocytes and NK cells [18]. In 1995, the porcine NK-lysin was firstly isolated from swine intestinal tissues [5]. Previous studies demonstrated that NK-lysin has a broad spectrum of antimicrobial activity including against bacteria, fungi, parasite, mycoplasma, and virus [19,20,21]. NK-lysin and its derivatives have been found to kill a variety of tumor cells, but harmless to erythrocytes [22,23,24,25]. Meanwhile, more and more evidence shows that AMPs can be used as key factors in immune regulation besides antibacterial and antiviral activities [26]. A previous study indicated that the AMPs can also act as an immunomodulator for mammals [8]. For instance, the porcine β-defensin (PBD) 2 was found to attenuate inflammation and mucosal lesions in dextran sodium sulfate-induced colitis [9].

In the present study, we describe the cloning and expression of PNKL gene by pET expression vector in *E. coli* Rosetta (DE3) by using a heterologous expression system. Moreover, the antimicrobial and anti-inflammatory activities of the PNKL protein were fully explored. This study not only reveals novel functions of the AMPs, but will also facilitate the development of substitutes for conventionally using antibiotics or drugs.

## 2. Results

### 2.1. Comparison of the PNKL Nucleotide Sequences

**Figure 1:**
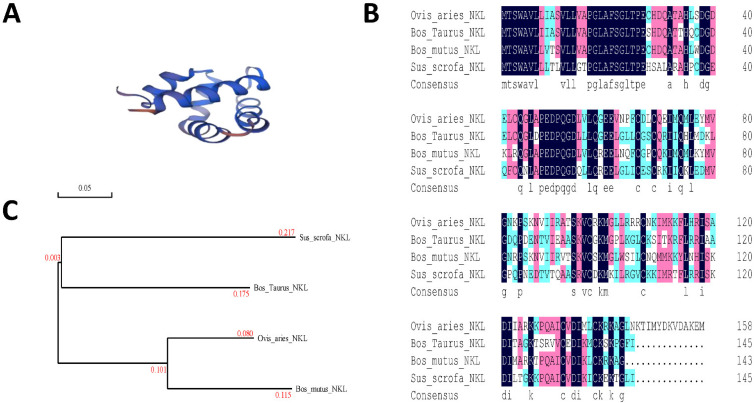
Phylogenetic analysis of Porcine NK-Lysin. (**A**), prediction model of PNKL; (**B**), phylogenetic analysis of PNKL in *Sus scrofa*, *Ovis aries*, *Bos Taurus*, and *Bos mutus* were performed by DNAMAN 8.0; (**C**), amino acid sequences of PNKL in *Sus scrofa*, *Ovis aries*, *Bos Taurus*, and *Bos mutus* were aligned by DNAMAN 8.0.

Blast analysis of the synthesized PNKL sequence was performed by using the DNAMAN 8.0. Results showed that the synthesized PNKL sequence was consistent with the published sequence (Supplementary Materials Figure S1). Both contained a 390-bp open reading frame, which encoded a 145-amino acid PNKL mature protein. Structural analysis using the “SWISS model” showed that the PNKL protein was composed of 5 alpha helices (Figure 1A). Amino acid sequence analysis showed that the amino acid sequence of PNKL was highly conserved (Figure 1B). The Porcine NK-Lysin sequence was more than 70.75% identical to the sequences obtained from *Ovis aries*, *Bos Taurus*, and *Bos mutus.* Phylogenetic tree analysis showed that the Porcine NK-Lysin was close to *Ovis aries* (Figure 1C).

### 2.2. Expression and Purification of the Recombinant PNKL

**Figure 2:**
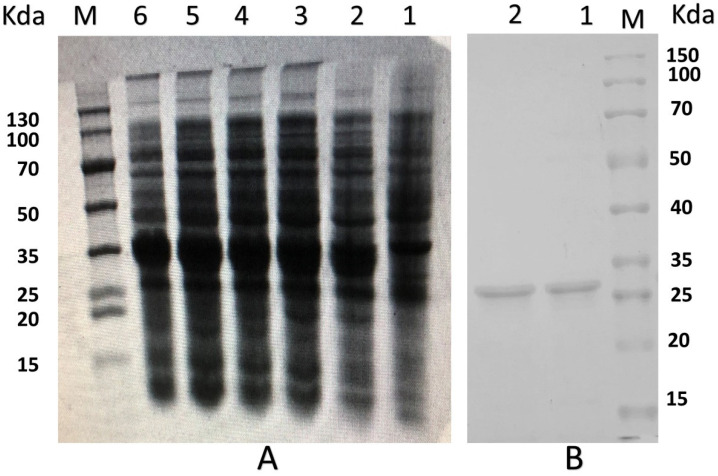
SDS-PAGE analysis of PNKL produced by *E. coli* Rosetta. (**A**), SDS-PAGE of PNKL from *E. coli* Rosetta. M protein markers (DL 130 kDa), Lane 1, *E. coli* Origami B (DE3)-pET32a (+) (non-induced), Lane 2, *E. coli* Origami B (DE3)-pET32a (+) induced by 1 mmol/L IPTG for 2 h at 28 °C, Lane 3, *E. coli* Origami B (DE3)-pET32a (+) induced by 1 mmol/L IPTG for 4 h at 28 °C, Lane 4, *E. coli* Origami B (DE3)-pET32a (+) induced by 1 mmol/L IPTG for 6 h at 28 °C, Lane 5, *E. coli* Origami B (DE3)-pET32a (+) induced by 1 mmol/L IPTG for 8 h at 28 °C, Lane 6, *E. coli* Origami B (DE3)-pET32a (+) induced by 1 mmol/L IPTG for 10 h at 28 °C; (**B**), Purification of PNKL. M protein markers (DL 150 kDa), Lane 1-2 PNKL purified by Ni 2+ -IDA affinity chromatography.

The PNKL gene with two designated restriction enzyme sites (EcoRI/NotI) was artificially synthesized. The two restriction enzyme sites allowed directional cloning of the PNKL gene into the pET32a expression vector. A 404-bp fragment was observed after double digestion of recombinant plasmid with the two restriction enzymes (Figure S2A). The recombinant plasmids were transformed into the *E. coli* Rosetta (DE3) and the positive clones were selected by PCR (Figure S2B). The most desired strain was chosen for small-scale induction by using 0.5 mmol/L IPTG at 28 °C. We found that the induction time significantly affected the expression level. As shown in Figure 2A, the *E. coli* achieved a maxima yield of the PNKL protein after 6 h induction (more than 250 μg/mL). The molecular weight of recombinant PNKL was estimated by SDS-PAGE to be 33 kDa (Figure 2A,B). The crude protein collected from ultrasonically-disrupted bacteria was purified by using Ni2^+^-IDA affinity chromatography (Figure 2B).

### 2.3. Antibacterial Activity of the PNKL

The antibacterial activities of PNKL was investigated by using Gram-negative and Gram-positive bacteria strains. As shown in Table 1, PNKL showed strong antibacterial activity against Gram-negative bacteria such as the *E. coli* K88 with a MIC of 4 µg/mL. PNKL also showed antibacterial activity against the *S. typhimurium* (with a MIC of 8 µg/mL). Moreover, the PNKL showed strong antibacterial activities against Gram-positive bacteria such as the *S. aureus* with a MIC of 2 µg/mL. PNKL also showed antibacterial activity against the *B. subtilis* (with a MIC of 8 µg/mL).

### 2.4. Hemolytic Activity of the PNKL

**Figure 3:**
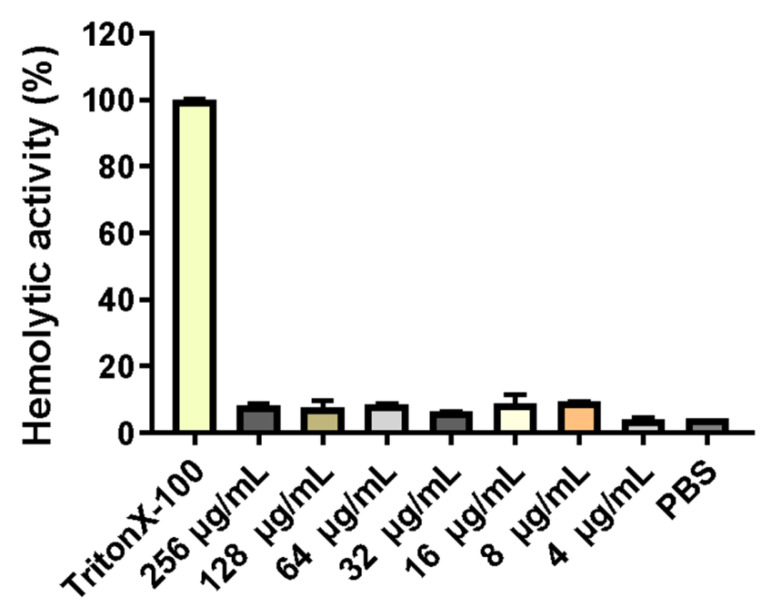
Hemolytic activity of recombinant of PNKL. PNKL 256 µg/mL PNKL, *Triton X-100 **0.1**%* Triton X-100, *PBS* 10 Mm PBS (pH 7.3).

Erythrocytes were collected from fresh porcine blood and incubated with different concentrations (0–256 mg/L) of PNKL for 1 h. As compared to the TritonX-100, the PNKL showed no significant hemolytic activity at all concentrations (Figure 3).

### 2.5. Effect of PNKL on Cell Viability, Apoptosis, and Localization of ZO-1 Protein in IPEC-J2 Cells Exposure to E. coli K88

**Figure 4:**
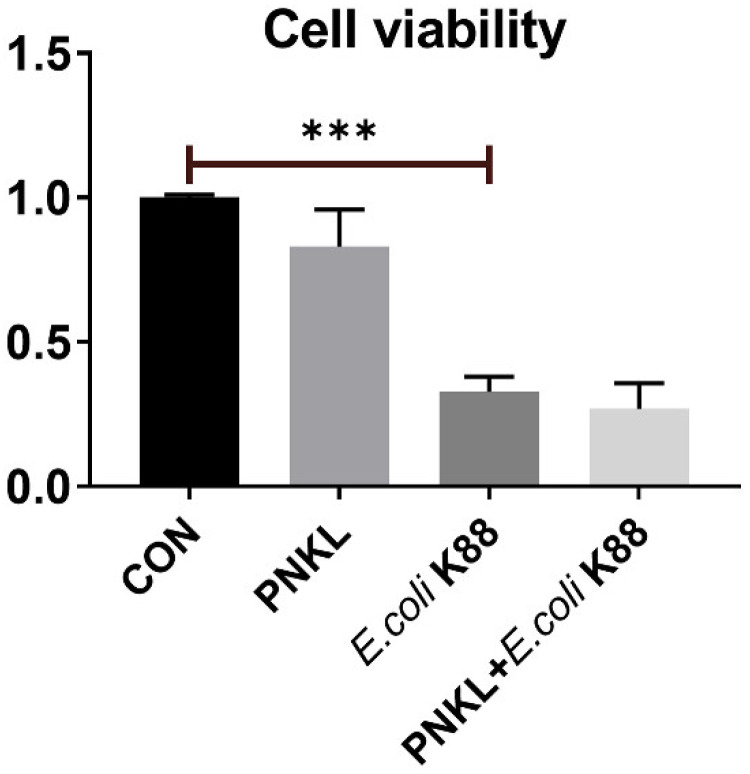
Influence of PNKL on *E. coli* K88-induced cell viability in IPEC-J2 cells. IPEC-J2 was determined by incubation with CCK8 for 1 h after different treatments. *** Means significant difference compared with blank control group (*p* < 0.001).

**Figure 5:**
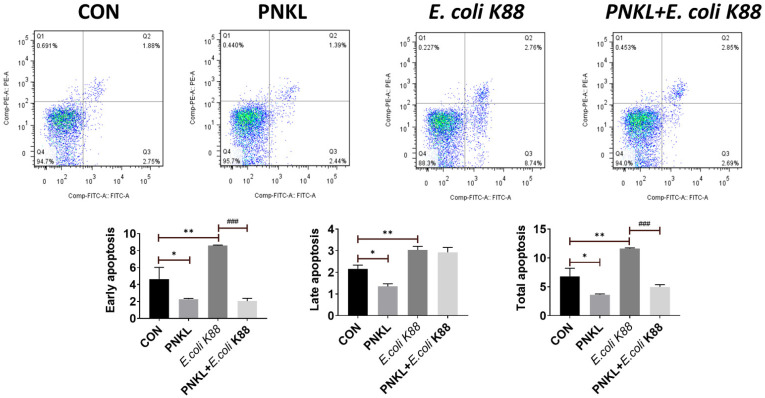
Influence of PNKL on *E. coli* K88-induced apoptosis in IPEC-J2 cells. Cell distribution analysis of apoptosis of IPEC-J2 cells treated with PNKL, *E. coli* K88, an PNKL plus *E. coli* K88. In each diagram, Q1 represents the percentage of non-viable, necrotic cells, Q2 represents the percentage of late apoptotic IPEC-J2 cells, Q3 represents the percentages of early apoptotic IPEC-J2 cells and Q4 represents the percentage of live IPEC-J2 cells. The statistical analysis of cell distribution data among samples, total apoptotic cells included Q2 with Q3. * Means significant difference compared with blank control group (*p* < 0.05), ** Means significant difference compared with blank control group (*p* < 0.01), ### Means significant difference compared with *E. coli* K88 control group (*p* < 0.001).

**Figure 6:**
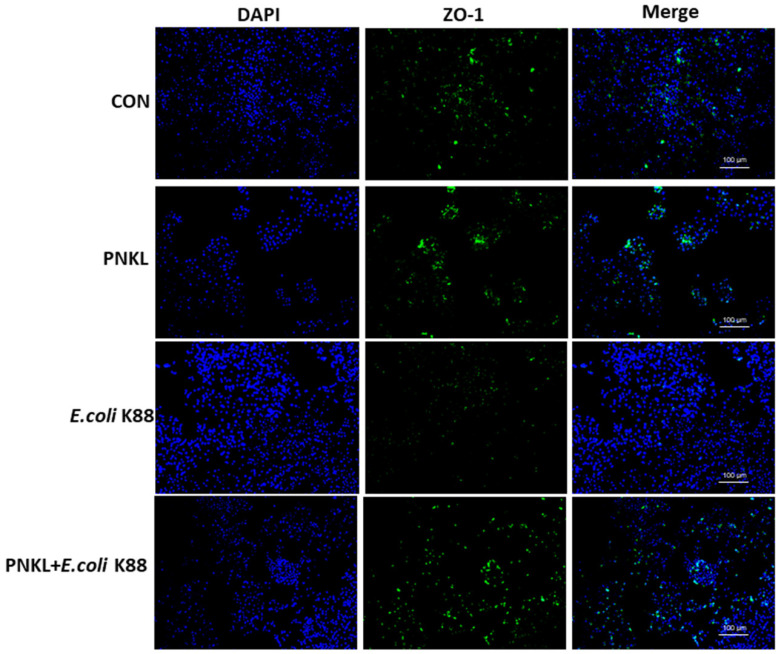
Effect of PNKL on distribution and abundance of ZO1 proteins. Representative immunofluorescent images for detection of ZO-1 (green) and DAPI (blue). Scale bar = 100 μm.

As shown in Figure 4, *E. coli* K88 challenge decreased the viability of the IPEC-J2 cells. However, PNKL treatment elevated the cell viability. As compared to the control group, *E. coli* K88 challenge significantly elevated the apoptosis rates in the IPEC-J2 cells (Figure 5). However, PNKL treatment significantly decreased the early apoptosis and total apoptosis rates in the *E. coli* K88-challenged cells (*p* < 0.05). Meanwhile, we explored the distribution and abundance of zonula occludens-1 (ZO-1), one of the major tight-junction-related proteins located in the intestinal epithelium, by immuno-fluorescence analysis. We found that the ZO-1 staining in IPEC-J2 was diffuse due to *E. coli* K88 challenge, with less staining in the tight intercellular junction area, indicating disruption of the tight junction upon *E. coli* K88 infection. However, PNKL treatment attenuated the *E. coli* K88-induced disruption by improving the localization and abundance of the ZO-1 proteins in IPEC-J2 cells (Figure 6).

### 2.6. Effect of PNKL on Expressions of Genes Involved in Inflammatory Response and Cell Apoptosis

**Figure 7:**
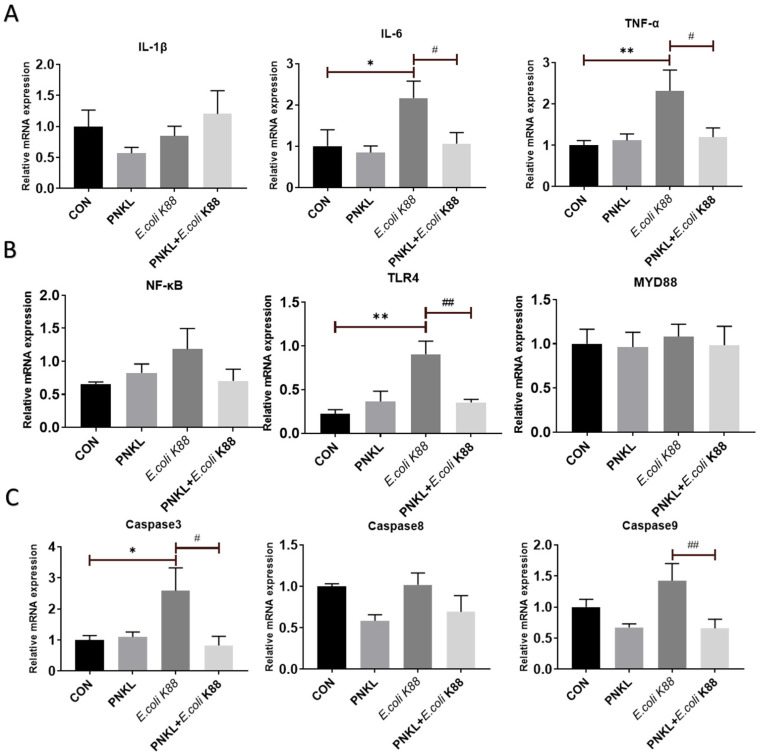
Influence of PNKL on *E. coli* K88-induced inflammatory responses in IPEC-J2 cells. Total RNA was extracted from IPEC-J2 cells and the expression of related genes were measured by real-time fluorescence PCR. The target gene mRNA expression level was calculated using the 2^–ΔΔCt^ method. (**A**) Gene expression of the NF-κB signaling pathway key factors. (**B**) Gene expression of the apoptotic factor. (**C**) Gene expression of the proinflammatory cytokine. * Means significant difference compared with blank control group (*p* < 0.05), ** Means significant difference compared with blank control group (*p* < 0.01), # Means significant difference compared with *E. coli* K88 control group (*p* < 0.05), ## Means significant difference compared with *E. coli* K88 control group (*p* < 0.01). IL-1β, interleukin 1 beta; IL-6, interleukin 6; TNF-α, tumor necrosis factor alpha.

In this study, *E. coli* K88 challenge significantly elevated the expression levels of inflammatory cytokines such as the IL-6 and TNF-α in the IPEC-J2 cells (Figure 7A). Moreover, *E. coli* K88 can induce the expression of NF-κB, and Toll-like receptor 4 (TLR4), the key proteins of the inflammatory signaling pathway, in which the expression of TLR4 is significantly increased (Figure 7B). However, PNKL treatment down-regulated their expression levels in the *E. coli* K88-challenged cells (*p* < 0.05). Moreover, PNKL treatment significantly down-regulated the expression level of caspase 3 and caspase 9 in the *E. coli* K88-challenged cells (Figure 7C) (*p* < 0.05).

## 3. Discussion

Antibiotic resistance is an imminent threat to the effective treatment of bacterial infections, and there is an urgent need for alternative antibiotic strategies [27]. The development of new therapeutic agents to combat bacterial infections should be prioritized [28]. The production cost of peptides is an important factor hampering the development of AMPs. The production of AMPs by recombinant expression methods using microorganisms resistant to the peptide produced is one economically viable and promising alternative [29]. NK-lysin is a cationic antibacterial that was originally isolated from porcine intestinal tissue based on its antibacterial activity [30,31]. Currently, the NK-lysin has attracted considerable research interest since it has been reported to show a broad-spectrum antimicrobial activity and participate in the regulation of immune functions [20]. More than 12 kinds of porcine antimicrobial peptides have been found, including porcine thrombin, β-defensin-2, cecropin P1, and NK-lysin, which have broad-spectrum bactericidal effect on many microorganisms [32,33]. Among them, NK-lysin can directly combine the lipid part of lipopolysaccharide, resist various bacteria and fungi, such as *E. coli*, *Bacillus megaterium*, *Acinetobacter acetate*, and even dissolve some tumor cells [18]. In the present study, the PNKL was obtained by using heterologous expression, and the recombinant PNKL was purified and fully characterized.

The recombinant PNKL was estimated by SDS-PAGE to be 33 kDa. It is well known that culture conditions such as induction time and temperature can affect protein expression in the heterogenous expression system [34]. We obtained the highest yield of PNKL after inducing *E. coli* with IPTG for 6 h. This is different from previous studies on the time when NK-lysin reaches its maximum expression [34]. The difference may result from the use of different bacteria strains. Interestingly, antimicrobial activity assays showed that PNKL has significant antimicrobial activity against both the Gram-positive bacteria (*S. aureus* and *B. subtilis*) and Gram-negative bacteria (*E. coli* K88 and *S. typhimurium*). The MIC for PNKL against *E. coli* K88 was 4 μg/mL. PNKL also showed antibacterial activity against *S. aureus* (with a MIC of 2 μg/mL), which is lower than NK-lysin obtained in the previous study [35]. These results are also consistent with a previous report on the PNKL, and both results indicated that the PNKL as well as its derivative had a broad-spectrum of antibacterial activities [24,36,37]. Importantly, hemolytic assays showed that PNKL had no detrimental impact on cell viability, similar to previous studies. This indicates that it is safe for human use and may be tentatively used as a substitute for conventionally used antibiotics [34].

In addition to its antibacterial activity, previous study suggests that PNKL is involved in the body’s immune regulation [38]. For instance, chicken NK-lysin, fish NK-lysin, and salmo salar cathelicidin 1 were found to have both the anti-inflammatory and immunoregulatory functions [39,40,41,42,43]. Therefore, we further investigated the influence of PNKL on the inflammatory response of intestinal epithelial cells exposed to *E. coli* K88. We found that *E. coli* K88 challenge significantly decreased the cell viability and elevated the apoptosis rate in the IPEC-J2 cells. Our results are similar to those of previous studies in that microbial infection or stress can increase the apoptosis of intestinal epithelial cells [44]. Interestingly, PNKL significantly decreased the apoptosis in the *E. coli* K88-challenged cell. This is probably due to the down-regulation of several critical inflammatory cytokines such as the IL-1β and TNF-α. Inflammatory cytokines, including IL-6, IL-1β, and TNF-α, are cytokines involved in inflammation, which could be released quickly under pathological conditions, leading to changes in systemic metabolism and disruption of the tissues such as the muscle and intestinal mucosa [45,46,47].

Tight junctions are composed of occludin, claudins, and zonula occludes (Zo)-1, -2, -3, which control the cell bypass permeability of endothelial cells and epithelial cells and provide barrier functions, such as inhibiting bacterial invasion [48]. However, various enteric pathogens can cause intestinal epithelial permeability defects by altering the distribution of tight junction proteins [49,50]. Previous studies indicated that *E. coli* infection elevated the mRNA and protein levels of tight junction proteins ZO-1 and occludin [51,52]. PNKL treatment attenuated the *E. coli* K88-induced mucosa lesion. In our study, the ZO-1 staining in the IPEC-J2 cells was diffuse with little staining at the intercellular tight junction region in the *E. coli* K88-challenged IPEC-J2 cells, indicating disruption of the TJs upon *E. coli* K88 infection. However, PNKL treatment attenuated the ETEC-induced TJs disruption by improving the localization and abundance of the ZO-1 proteins. As the bacterial endotoxins (i.e., lipopolysaccharide) and inflammatory cytokines (i.e., TNF-α) are detrimental to intestinal epithelial cells, they induce mucosal damage [53,54]. Therefore, we speculate that PNKL may improve mucosal morphology and tight junction due to its antibacterial and anti-inflammatory activities.

In order to further understand the mechanism of PNKL regulating intestinal barrier function, we explored the expression level of some critical molecules involved in inflammatory response and apoptosis regulation. Enteropathogenic bacteria (ETEC) adhere to the intestinal epithelium and cause severe diarrhea and intestinal inflammation through lipopolysaccharide (LPS), the main component of the cell wall [55]. LPS cannot directly destroy the integrity of cell structure and activate the signal pathway of apoptosis, but it can bind to TLR4, activate NF-κB, signal pathway, and produce the inflammatory factor (i.e., TNF-α) [56]. In the present study, an *E. coli* K88 challenge significantly elevated the expression level of TLR4 in the IPEC-J2 cells. Elevated TLR4 expression activates the NF-κB signaling pathway to release inflammatory cytokines. Many cationic peptides inhibit the proinflammatory response by reducing the proinflammatory mediators (mainly TNF-α) [57,58]. Previous studies have found that Chicken NK-lysin derived peptide cNK-2 can be used as an immunomodulator to regulate TLR agonist-induced inflammatory response [59]. In this study, *E. coli* K88 challenge significantly elevated IL-6 and TNF-α expression levels in the IPEC-J2 cells. However, PNKL treatment resulted in significant down-regulation of the two critical inflammatory cytokines. Therefore, we speculate that PNKL may inhibit the NF-κB signaling pathway by inhibiting the expression of TLR4, resulting in decreased secretion of inflammatory cytokine. The specific mechanism needs to be further studied.

Caspases are an evolutionarily conserved family of cysteine proteases, mainly involved in cell death and inflammation [60]. The family of caspases can be functionally subdivided into initiators (caspases 8, 9, 10) and effectors (caspases 3, 6, 7) [61]. Among these executioners, caspase 3 is a cysteine–aspartic acid protease that cleaves cellular targets and executes cell death [62]. In this study, an *E. coli* K88 challenge significantly elevated the expression levels of caspase 3 in the IPEC-J2 cells, which was consistent with previous study on piglets [63]. However, PNKL can down-regulate the expression levels of caspase 3 and caspase 9. This is probably due to the down-regulation of inflammatory cytokines (i.e., IL-6 and TNF-α) since they were reported to induce apoptosis via activation of the caspase system [64,65].

The PNKL shows a broad-spectrum of antimicrobial activities against both the Gram-negative and Gram-positive bacteria. However, it shows only a little light hemolytic activity and cytotoxicity. Moreover, PNKL increases the cell viability and attenuates the inflammatory response and injury in intestinal epithelial cells exposured to *E. coli* K88, which was associated with decreased cell apoptosis and down-regulation of inflammatory cytokines. The anti-bacterial and anti-inflammatory properties of PNKL may allow it to be a potential substitute for conventionally used antibiotics or drugs. However, there are still some unclear mechanisms, which need further exploration.

## 4. Materials and Methods

### 4.1. Strains and Vectors

The *E. coli* DH5α and *E. coli* Rosetta (DE3) strains were purchased from TIANGEN (Beijing, China). *E. coli* K88 was kindly provided by Professor Lianqiang Che, Institute of Animal Nutrition, Sichuan Agricultural University. *Salmonella typhimurium* ATCC14028 (*S. typhimurium*), *Staphylococcus aureus* CICC23656 (*S. aureus*), and *Bacillus subtilis* (*B. subtilis*) were kindly provided by Professor Qigui Yan, College of Animal Science and Technology, Sichuan Agricultural University. The pET32a (+) was purchased from Invitrogen.

### 4.2. Plasmid Construction

PNKL gene was obtained by complete gene synthesis (Beijing Cycle-Tech Biotechnology Co., Ltd., Beijing, China). The company provides a cloned strain of DH5α-PMD19-PNKL containing the target gene porcine NK-lysin (PNKL). Sequencing identification was. Performed at ShengGong Biological Engineering Co., Ltd. Sequencing results were compared by software DNAMAN. The plasmid PMD19-PNKL was extracted by using Plasmid Mini Kit I (Omega, Norcross, GA, USA) according to the manufacturer’s recommendations. The plasmid pMD19-PNKL and expression vector pET32a (+) were double-digested with EcoRI and NotI Enzymes (Takara, Otsu, Japan) at 37 °C for 4 h. After purification by agarose gel electrophoresis, the isolated DNA fragments were ligated by T4 DNA ligase (Takara, Otsu, Japan). Then, the ligated product was transformed into *E. coli* DH5α cells using the heat shock method and plated on LB agar containing kanamycin (50 µg/mL). The positive colonies were randomly picked, then confirmed by restriction enzyme digestion and sequenced by Sangon Biotech (Shanghai, China).

### 4.3. Inducible Expression of PNKL

For expression, plasmids pET32a (+)-PNKL gene were transformed into *E. coli* Rosetta (DE3) cells using heat shock method. Positive bacterial colonies were then confirmed as the methods of construction of the expression vector. The selected positive bacterial were incubated. Once OD_600_ reached 0.8, 1.0 mM isopropyl β-d-1-thiogalactoside (IPTG) was added to induce protein expression. After incubation for 6 h at 28 °C, bacterial cells were harvested by centrifugation at 8000× *g* for 10 min at 4 °C and lysis by lysis buffer (500 mM NaCl, 20 mM Tris, 0.1% Triton X-100, 1 mM PMSF, Lysozyme 0.2 mg/mL, 10 U/mL DNase (pH 7.5)) for 30 min at 4 °C. Schizolytic cells were then sonicated (4 s pulse and 8 s interval; 30 cycles; Sonics-Vibra cell). They were then centrifuged at 15,000× *g* for 30 min at 4 °C and the supernatant was run on 12% SDS-PAGE or for affinity purification.

### 4.4. Affinity Purification

The supernatant obtained above was filtered by 0.22 µm filter, and then applied to Ni 2^+^-IDA column (Sangon Biotech, Shanghai, China) and purified according to the manufacturer’s specifications. Briefly, 10 resin volumes of Binding Buffer (50 mM NaH_2_PO_4_, 300 mM NaCl, pH 8.0) was added to wash way the impure protein, then 5 resin volumes of Elution Buffer (50 mM NaH_2_PO_4_, 300 mM NaCl, 150 mM Imidazole, pH 8.0) was added to elute the PNKL from the column. The protein concentration was quantified with the BCA assay (Beyotime, Shanghai, China). The purified PNKL was run on 12% SDS-PAGE. The rest was stored at −80 °C to analyze antimicrobial activities.

### 4.5. Antimicrobial Activity Assays

The minimal inhibitory concentration (MIC) of purified PNKL was measured by the microtiter broth dilution method [66]. *E. coli* DH5α, pathogenic *E. coli* K88+, *S. typhimurium*, *S. aureus*, and *B. subtilis* were grown to 0.4 OD_600_ nm at 37 °C in LB, *Streptococcus* was grown to 0.4 OD_600_ nm at 37 °C in THY (Todd-Hewitt + yeast extract). The target cell culture was diluted to 1 × 10^5^ CFUs/mL with the same media respectively. A total of 100 µL of PNKL and 100 µL of cell suspension were added into each well. The activity of PNKL was tested over a concentration range of 256, 128, 64, 32, 16, 8, 4, 2, and 1 µg/mL, and all assays were tested in triplicate. Bacterial plates were incubated at 37 °C for 16 h, and the absorption of cell culture was recorded at 600 nm. MIC was defined as the lowest concentration of peptide at which there was no change in optical density.

### 4.6. Hemolytic Activity Assay

The hemolytic activity of PNKL was measured spectrophotometrically using a hemoglobin release assay [67]. Fresh pig blood was collected in a heparinized-tube and centrifuged at 1500 rpm for 10 min. This study was approved by the Animal Welfare Committee of Sichuan Agricultural University (No. 20180718). The pellet was gently washed three times with cold PBS (pH 7.2), and resuspended in cold PBS (pH 7.2), then the erythrocytes concentration was adjusted to 4%. A 200 µL erythrocyte suspension was added to a 96-well microtiter plate. Different concentrations of NK-lysin solution were added to each well and incubated for 60 min at 37 °C. Triton-X 100 (0.1%) and PBS were used as positive and negative controls, respectively. The release of hemoglobin of the supernatant was measured after centrifugation (1000 rpm for 5 min) by UV-1100 spectrophotometer (Shanghai, China) at 414 nm. No hemolysis and 100% hemolysis were determined in PBS and Triton X-100, respectively.

### 4.7. Cell Culture

Intestinal porcine epithelial cells (IPEC-J2) were cultured in a 75 cm^2^ cell culture flask in DMEM-F12 with 10% FBS, 100 U/mL penicillin, and 100 μg/mL streptomycin. Next, 1 × 10^5^ cells/well were seeded in 12-well plates and grown to ~60% confluence at 37 °C in a CO_2_ incubator (5% *v*/*v*), then incubated with antimicrobial peptides PNKL for 24 h (PNKL, PNKL+*E. coli* K88). Cells were challenged with 1 × 10^6^ CFU/well *E. coli* K88 for 1 h (PNKL+*E. coli* K88, *E. coli* K88), and control cells were cultured in a culture medium of 2% serum (without any antibiotics) without any treatment. Total cellular RNA was collected using RNAiso Plus (Takara, Dalian, China).

### 4.8. Cytotoxicity Assay

The cytotoxicity of PNKL was measured according to a previous study [68]. Briefly, IPEC-J2 cells were cultured in DMEM-F12 with 10% FBS, 100 U/mL penicillin, and 100 μg/mL streptomycin for 48 h and then resuspended to 10^5^ cells/mL in FBS free DMEM-F12 media. A volume of 100 μL of cells was aliquoted into sterile flat-bottomed 96-well plates (Corning, New York, NY, USA). The PNKL was added to the cells and incubated at 37 °C/5% CO_2_ for 24 h (PNKL, PNKL+*E. coli* K88). Then cells were challenged with 1 × 10^6^ CFU/well *E. coli* K88 for 1 h (PNKL+*E. coli* K88, *E. coli* K88), control cells were cultured in complete medium without any treatment. Cell viability was evaluated with the CCK-8 assay (Beyotime, Shanghai, China) according to the manufacturer’s instructions.

### 4.9. Assessment of Apoptosis by Flow Cytometry

The proportion of apoptotic cells in IPEC-J2 cells was determined by flow cytometry (CytoFlex, Beckman Coulter, Inc., Brea, CA, USA) using PE Annexin V Apoptosis Detection Kit I (Becton, Dickinson and Company, BD Biosciences, San Jose, CA, USA). When IPEC-J2 cells were grown to ~60% confluence at 37 °C in a CO_2_ incubator (5% *v*/*v*), they were incubated with PNKL for 24 h (PNKL, PNKL +*E. coli* K88). Cells were challenged with 1 × 10^6^ CFU/well *E. coli* K88 for 2.5 h (PNKL+*E. coli* K88, *E. coli* K88) before sample collection, and control cells were cultured in a culture medium without any treatment. Treated cells were harvested and labeled with an anti-Annexin V-FITC Apoptosis Detection Kit (BD Biosciences, San Jose, CA, USA). Floating cells were collected, then attached cells were washed with 0.01 M PBS and digested with trypsin for 2 min. Finally, the digested cells and floating cells were added together to centrifugate at 350× *g* for 10 min, then the cells were stained with 2 μL of Annexin-V FITC fluorescent dye at 4 °C in the dark. After 10 min, 2 μL of PI staining was added for 5 min at 4 °C in the dark. Finally, detection of apoptotic cells was completed within 1 h after the addition of 400 μL Annexin V binding buffer (1×).

### 4.10. RNA Extraction and RT-PCR

IPEC-J2 cells were harvested and the total RNA was extracted using RNAiso Plus (Takara, Dalian, China) according to the manufacturer’s instructions. The quantity and quality of the isolated RNA were determined by absorbance at 260 and 280 nm [69]. cDNA was synthesized using a Reverse Transcriptase kit (Takara, Dalian, China). Briefly, quantitative PCR was performed by QuanStudio 6 Flex Real-Time PCR detection system (Applied Biosystems, Foster City, CA, USA) with a total of 10 µL of assay solution containing 5 µL SYBR Green mix (Takara), 0.2 µL Rox, 3 µL deionized H_2_O, 1 µL cDNA template, and 0.4 µL each of forward and reverse primers (Qingke, Beijing, China). The relative gene expressions compared with the housekeeping gene β-actin were calculated by 2^−CT^ [70].

### 4.11. Immunofluorescence

IPEC-J2 cells were cultured in DMEM-F12 with 10% FBS, 100 U/mL penicillin, and 100 μg/mL streptomycin for 48 h and then resuspended to 10^5^ cells/mL in FBS free DMEM-F12 media. A volume of 1 mL of cells was aliquoted into sterile flat-bottomed 12-well plates (Corning, New York, NY, USA). The PNKL was added to the cells and incubated at 37 °C/5% CO_2_ for 24 h (PNKL, PNKL+*E. coli* K88). Cells were challenged with 1 × 10^6^ CFU/well *E. coli* K88 for 1 h (PNKL+*E. coli* K88, *E. coli* K88), and control cells were cultured in complete medium without any treatment. We added 300 μL of fixative solution (4% paraformaldehyde) into each well and left them to stand for 15 min at room temperature. Each well was washed three times with PBS (pH 7.4) for 2 min each time, and then samples were incubated overnight with the primary antibody at 4 °C and kept in the dark (rabbit anti-ZO-1; Novus). The samples were washed three times with PBS (pH 7.4) for 2 min each time and incubated for 2 h at room temperature with the appropriate secondary antibody (Alexa Fluor 488 conjugated goat anti-rabbit immunoglobulin (CST)). Samples with the biotinylated secondary antibody were incubated with Avidin Alexa Fluor 488 (CST) for visualization of the secondary antibody. Samples were washed, and nuclei were counterstained with DAPI (Sigma-Aldrich, St. Louis, MO, USA). Samples were imaged using a leica inverted microscope (DMI4000B).

### 4.12. Statistics Analysis

All statistical analysis was performed using SPSS 21.0 software. Data were expressed as the mean ± standard error of the mean (SEM). Statistical analysis of treatment of IPEC-J2 and cytotoxicity were carried out using two-way ANOVA followed by Duncan’s multiple comparisons test. Image production was performed using GraphPad Prism software (Version 7. GraphPad Software Inc., San Diego, CA, USA).

## Figures and Tables

**Table 1:** MIC of PNKL produced by *E. coli* Rosetta (DE3).

Strain	MIC (µg/mL)
Gram-negative bacteria	
*E. coli* DH5α 32	32
Pathogenic *E. coli* K88	4
*Salmonella typhimurium* CICC14028	8
Gram-positive bacteria	
*Streptococcus*	16
*Staphylococcus aureus* CICC23656	2
*Bacillus subtilis*	8

## Data Availability

The datasets during and/or analyzed during the current study are available from the corresponding author on reasonable request.

## Supplementary Materials

The following are available online, Figure S1: Strategy of cloning of PNKL gene. The CDS sequence without signal peptide of PNKL was cloned, EcoR Ι and Not Ι was insert in 5′ and 3′ respectively, Figure S2: Results from agarose gel electrophoresis (EcoR I/Not I digestion map). (A) Digestion of the recombinant expression vectors. Lane M1 DNA marker (DL 10,000), Lanes 1 PNKL (without digestion). Lanes 2 PNKL (digestion with EcoR I and Not I) Lane M2 DNA marker (DL 2000). (B) RT-PCR of PNKL. Lane M DNA marker (DL 4000), Lanes 1-2 PNKL (production of five tubes of reaction solution).

## Abbreviation

MIC	minimal inhibitory concentration
