# Supplementary material for: Functional Characterization of Porcine NK-Lysin: A Novel Immunomodulator That Regulates Intestinal Inflammatory Response
Source: Molecules. 2021 Jul 13;26(14):4242. doi: 10.3390/molecules26144242 (PMC8307250; doi:10.3390/molecules26144242)

Additional file:

Fig. S1 Strategy of cloning of PNKL gene.

The CDS sequence without signal peptide of PNKL was cloned, *EcoR* I and *Not*I was insert in 5’ and 3’ respectively.

EcoR I

GAATTCCCAGGGCTGGCCTTTTCCGGTCTGACCCCTGAGCA
CTCTGCCCTGGCAAGGGCCCACCCATGCGACGGAGAGCAG
TTCTGCCAGAACCTGGCCCCGGAGGACCCCCAGGGTGACC
AGCTGCTCCAAAGAGAGGAGCTGGGCCTCATCTGTGAGTCT
TGTCGGAAGATAATCCAGAAGCTGGAGGACATGGTGGGACC
ACAACCCAACGAGGACACTGTCACCCAGGCAGCCTCCCGG
GTGTGTGACAAGATGAAGATACTGAGAGGTGTGTGCAAGA
AGATCATGAGGACCTTTCTCCGTCGCATCTCCAAGGACATCC
TGACTGGGAAGAAACCCCAGGCTATCTGTGTGGACATCAAG
ATCTGTAAAGAGAAGACAGGTCTCATCTGA***GCGGCCGC***

Not I

Fig. S2 Results from agarose gel electrophoresis (EcoRI/Not I digestion map).

A Digestion of the recombinant expression vectors. Lane M_1 DNA marker (DL 10 000), Lanes 1 PNKL (without digestion).Lanes 2 PNKL (digestion with EcoRI and NotI) Lane M_2 DNA marker (DL 2000).b RT-PCR of PNKL. Lane M DNA marker (DL 4000), Lanes 1-2 PNKL (production of five tubes of reaction solution).

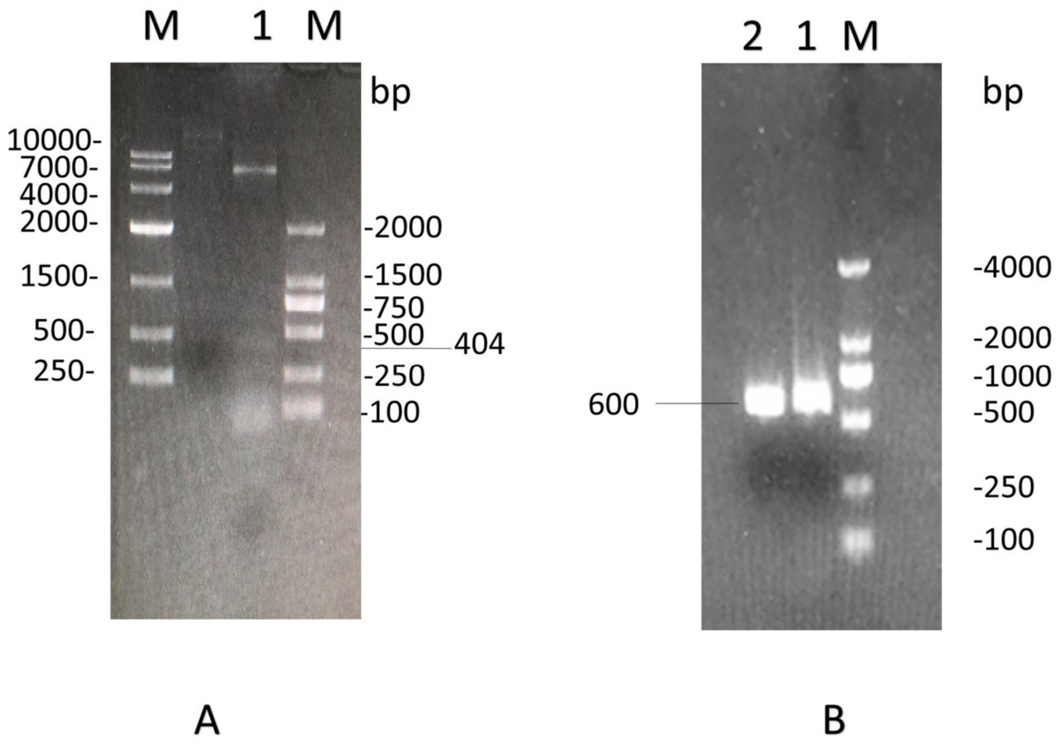

Supplement: Supplementary file 1 [file molecules-26-04242-s001.zip › molecules-1283511-supplementary.pdf]
